# Effects of Black Quinoa Wet-Milling Coproducts on the Quality Properties of Bologna-Type Sausages During Cold Storage

**DOI:** 10.3390/foods9030274

**Published:** 2020-03-03

**Authors:** Juana Fernández-López, Raquel Lucas-González, Alba Roldán-Verdú, Manuel Viuda-Martos, Estrella Sayas-Barberá, Jaime Ballester-Sánchez, Claudia Monika Haros, José Angel Pérez-Álvarez

**Affiliations:** 1 IPOA Research Group, Agro-Food Technology Department, Escuela Politécnica Superior de Orihuela, Universidad Miguel Hernández de Elche, Orihuela, 03312 Alicante, Spain; raquel.lucas@umh.es (R.L.-G.); alba.roldan@umh.es (A.R.-V.); mviuda@umh.es (M.V.-M.); estrella.sayas@umh.es (E.S.-B.); 2 Cereal Group, Instituto de Agroquímica y Tecnología de Alimentos (IATA-CSIC), 46980 Valencia, Spain; jballester@iata.csic.es (J.B.-S.); cmharos@iata.csic.es (C.M.H.); ja.perez@umh.es (J.A.P.-Á.)

**Keywords:** black quinoa, meat product, storage, shelf-life, wet-milling

## Abstract

The incorporation of a new ingredient into foods could not only affect the intrinsic properties of the product but also its shelf life. The aim of this study was to investigate the effect of the black quinoa (both as whole seeds and as the fibre-rich fraction obtained as coproduct from its wet-milling process) on the shelf life of Bologna-type sausages during cold storage. Three treatments of Bologna-type sausages were produced: control, sausages with black quinoa seeds (2.5%), and sausages with their fibre-rich fraction (2.5%). The effect of the black quinoa added on the physicochemical properties (pH and colour), lipid oxidation, residual nitrite level, and microbiological quality of Bolognas during 21 days of cold storage was evaluated. Although the addition of quinoa products in Bologna-type sausages modified some colour parameters (day 0), these differences were masked through the storage period. Sausages with quinoa products added showing lipid oxidation values lower than the control for all the days studied. Sausages with quinoa products added showed higher residual nitrite levels than control at all measurement times during the storage period. The addition of black quinoa did not affect microbial stability during storage. Black quinoa products can be considered promising ingredients to be used as antioxidants and natural nitrate sources in Bologna-type sausages without affecting their microbial safety during storage.

## 1. Introduction

Bologna-type sausages prepared by using a mixture of meat, fat, salt, and additives and heat-treated are publicly eaten as a meat foodstuff in numerous territories. Their production and consumption have been identified with different and diverse cultures around the world dating back many centuries. Sausage manufacture is a simple process of allowing the meat to undergo a series of controlled structural and chemical changes. These are basic to all cultures, but the changes rely on varied methods of preparation and spicing and recently also the addition of some non-meat ingredients with healthy properties or the substitution of additives by natural compounds, to achieve desired distinctive characteristics [1,2,3]. Even though the size and scope of operation have undergone a remarkable level of change, the principles and idea behind modern-day sausage manufacture in achieving products of high organoleptic value and improved shelf life remain the same [4].

In this context, quinoa (*Chenopodium quinoa*), although previously considered a food of low social prestige, currently has aroused great interest worldwide as an ingredient with potential health benefits and exceptional nutritional value: high concentration of protein (all essential amino acids highly bioavailable), unsaturated fatty acids, a low glycaemic index; vitamins, minerals, and other beneficial compounds; it is also gluten-free; furthermore, quinoa is a sustainable food, as plants exhibit a carbon and water footprint that is between 30 and 60 times lower than that of beef [5,6]. All these properties attributed to quinoa have made it a very attractive ingredient to be incorporated into different foods [7,8]. Currently, there is also a growing interest in the food industry to obtain high-added-value products from quinoa grains, by isolating some of their chemical components such as starch, fibre, protein, and oil, among others [9,10]. The wet-milling methods are usually involved in the processes implemented to obtain the purest possible fraction of each component (mainly starch), generating several quinoa coproducts (the fibre-rich or protein-rich fractions) [9] that could be used for technological and food purposes. 

In the meat industry, quinoa products (seeds, flour, and their coproducts) have been used in different meat products not only as a fat replacer, for its nutritional and healthy properties [11,12], but also for its technological properties, mainly as extender or binder [13,14,15,16,17,18], gelling agent [19], or even as frozen protected agent [20]. Most of these studies are focused on the new products’ characterization without assessing the impact of this new ingredient on the meat product’s shelf-life. Therefore, the aim of this study was to investigate the effect of the black quinoa (both as whole seeds and as the fibre-rich fraction obtained as coproduct from its wet-milling process) on the shelf life of Bologna-type sausages during cold storage. 

## 2. Materials and Methods 

### 2.1. Plant Materials

Black Bolivian Real quinoa seeds (*C. quinoa*) (BQS) from organic farming were supplied by a local supermarket in Orihuela (Alicante, Spain). The fibre-rich fraction of black quinoa seeds (RFBQ) was obtained as a coproduct from its wet-milling, following the Ballester-Sánchez et al. [9] methodology. 

### 2.2. Preparation of Bologna-Type Sausages

Three treatments were manufactured to determine the effect of black quinoa products on the shelf life of bologna-type sausages. Treatments were prepared in the food pilot plant at Miguel Hernández University according to the formulation showed in Table 1 and following normal industrial processing methods. Pork meat and pork backfat were obtained from a local supermarket (Orihuela, Alicante, Spain). The rest of the additives and spices were served by River (Orihuela, Alicante, Spain) a company dedicated to the supply and equipment for the food industry. 

Bologna-type sausages were manufactured following the procedure described by Fernández-López et al. [18]. Briefly, lean pork meat and salt were chopped in the cutter (R 2 Robot-Coupe, Robot-Coupe S.N.C., Vincennes Cedex, France) to favour the extraction of myofibrillar proteins. After that, the remaining ingredients, additives and pork backfat were added. This batter was stuffed in water-impermeable plastic casing (Fibran-Pack, Fibran, Girona, Spain) 80 mm in diameter. Sausages were cooked in a water bath until 72 °C was reached in the inner part of the product. After cooking, they were immediately cooled in an ice bath. The sausages were vacuum-packed in low-density polyethylene bags and stored at 4 °C for 21 days. The quality property analyses were performed during storage at 0, 7, 14, and 21 days.

### 2.3. Physicochemical Analysis 

The pH was measured in triplicate, directly using a Hach puncture electrode probe (5233) connected to a pH-meter (model SensION TM + pH3, Hach-Lange S.L.U., Vésenaz, Switzerland). The colour parameters (CIELAB Colour Space) of sausages were measured using a Minolta CM-700 (Minolta Camera Co., Osaka, Japan) spectrophotometer with illuminant D65, 10° observer, 11 mm aperture of the instrument for illumination and 8 mm for measurement [21]. Three sausages from each formulation were sliced (2 cm thickness) to avoid colour from being affected by the background and the colour parameters were evaluated on the internal surface at 3 different points. The following colour coordinates were determined: lightness (L*), redness (a*, +/– red–green), and yellowness (b*, +/– yellow–blue). The chroma saturation index (C* = (a*^2^ + b*^2^)^1/2^), the hue angle (H* = tan^−1^ b*/a*), and the colour differences (ΔE* = (ΔL*^2^ + Δa*^2^ + Δb*^2^)^1/2^) with respect to day 0, were also estimated. The reflectance spectra at every 10 nm between 360 and 740 nm were also obtained. 

### 2.4. Residual Nitrite Level

Residual nitrite level (mg NaNO_2_/kg sample) was assessed in triplicate, following ISO/DIS 2918 standards (International Organization for Standardization/Draft International Standard) [22]. 

### 2.5. Lipid Oxidation 

The extent of lipid oxidation was measured by the 2-thiobarbituric acid (TBA) method [23]. Thiobarbituric acid reactive substances (TBARS values, expressed as milligrams of malondialdehyde per kg sample, were calculated from a standard curve of malondialdehyde. Three samples in each of the three replications of the experiment were analysed in triplicate.

### 2.6. Microbiological Analysis

A 25 g aliquot of each sample was aseptically obtained and homogenized with 225 mL of sterile 0.1% peptone water in a Stomacher 400 (Colworth, London, UK) for 1.5 min. Aliquots (serial decimal dilutions) were plated out following standard methodologies. Mesophilic aerobic bacteria (MAB), lactic acid bacteria (LAB), Enterobacteriaceae, and moulds and yeasts were determined by plating the diluted samples in 3M Petrifilm™ plates for total viable counts (modified standard methods nutrients, 37 °C/48 h), for lactic acid bacteria counts (modified standard methods nutrients, 37 °C/48 h), for Enterobacteriaceae (modified Violet Red Bile Glucose nutrients, 37 °C/24 h), and for moulds and yeasts (nutrients supplemented with antibiotics, 25 °C/5 days), respectively. Results were expressed as log CFU(colony forming units)/g. Three samples in each of the three replications of the experiment were analysed in triplicate.

### 2.7. Statistical Analysis

A randomized complete block design was selected, and the entire experiment was replicated 3 times on 3 different days. In this case, the experiment included a total of 36 observations (3 treatments × 4 storage time × 3 replications) for statistical analysis. Data were analysed by analysis of variance (ANOVA) considering the replicates as a random effect and the treatments and storage time as a fixed effect. Tukey test was used to analyse significant differences at a 5% significance level. All these analyses were made by the SPSS statistical program (SPSS, Chicago, IL, USA) for Windows.

## 3. Results

### 3.1. Physicochemical Analysis

As regards pH values (Table 2), neither the addition of quinoa products nor storage time had any statistically significant effect (*p* > 0.05) on this parameter. pH values for all sausages may be considered normal for this type of meat product [1,3,24].

Meat product colour is significant to consumer acceptance of products. Colour formation in cured meat products involves, basically, the reaction of endogenous pigments in muscle, essentially myoglobin (Mb), with nitric oxide (NO) from added nitrite. The nitrosated and typical red pigment, nitrosomyoglobin (NOMb) is called “cured meat pigment”, and it is converted to nitrosohaemochrome by the cooking process. This latter pigment has a pinkish colour, which is known to be stable with cooking [25]. The importance of meat product colour is also reflected in the fact that improving colour stability will influence shelf life by increasing the time that the product is visually acceptable to consumers at retail. The addition of non-meat ingredients to meat products frequently affects not only the colour of the final product [2,26,27] but also the colour evolution during the storage period and then their shelf-life [1,2,26,28]. In the present study, lightness, yellowness, and chroma at the beginning of the storage period were affected (*p* < 0.05) only when the fibre-rich fraction of quinoa was added (RFBQS), with these samples showing the lowest values. The addition of quinoa products decreased redness values and increased hue values, being these changes higher (*p* < 0.05) when the fibre-rich fraction of quinoa was added (RFBQS).

After 21 days of storage, there was no change in L*, a*, b*, and C* values for control sausages and sausages with quinoa seeds added (BQS), but there was a significant increase (*p* < 0.05) for all these colour parameters when the fibre-rich fraction of quinoa was added (RFBQS). Only in control sausages, the storage period had no influence on hue values, although it was decreased (*p* < 0.05) in both sausages with added quinoa products (BQS and RFBQS). It is important to note that although the addition of quinoa products in bologna-type sausages modified some colour parameters (at day 0), these differences were masked through the storage period, showing at the end of this period (day 21) similar colour parameters for all the treatments. It is also supported by the values of colour differences (with respect to the control sausages) because although at day 0, sausages with fibre-rich fraction quinoa added (RFBQS) showed higher (*p* < 0.05) colour differences than sausages with quinoa seeds added (BQS), these differences were not significant at the end of the storage period. So, it could be said that at the end of the storage period all the sausages showed a similar colour. It should be remembered here that only colour differences higher than 3 units are detected by the human eye [29].

**Figure 1:**
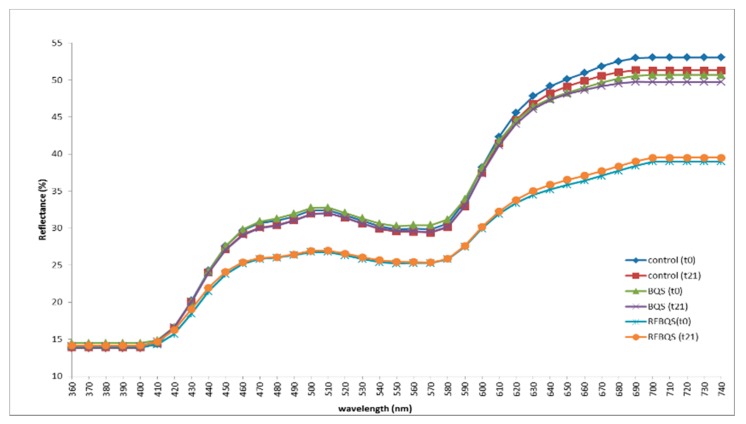
Reflectance spectra (360–740 nm) of the Bologna-type sausages (control, sausage with black quinoa seeds (BQS) and sausage with its fibre-rich fraction (RFBQS)) at the beginning (day 0) and at the end of the storage period (day 21).

The study of the reflectance spectra of the meat products could help to understand the subsequent changes in sausages due to both the application of new ingredients and storage conditions [18,30] comparing the shape and spectral intensity variations of one spectrum to those of others. Figure 1 presents the reflectance spectra (360–740 nm) obtained for the three bologna-type sausages (control, BQS, and RFBQS) at day 0 and at the end of the storage period (day 21). All the spectra corresponding to bologna-type sausages showed a similar shape, which has been described as typical for cooked cured meat products [18,21]. So, it can be said that neither the addition of quinoa products nor the storage period modified the typical reflectance spectrum shape for this type of meat product. On the contrary, both factors were responsible for some modifications in the reflectance intensity (%) at different wavelengths. The addition of quinoa products to bologna-type sausages caused changes in the reflectance intensity (%) for several wavelengths depending on the type of quinoa product added. When quinoa was added as whole seeds (BQS), the reflectance intensity decreased (*p* < 0.05) (with respect to that observed in the control sausage) only after 630 nm; when the fibre-rich fraction of quinoa was added (RFBQS), this decrease was significant at lower wavelengths (after 440 nm) and with higher intensity. Most of these changes could be related to the presence of colourant compounds in the black quinoa, mainly betacyanins [31] and their interferences with the nitrosohaemochrome, that has been previously reported as the main pigment responsible for the typical colour in cooked and cured meat products. Furthermore, these betacyanins are mainly located in the quinoa seed coat, so it was expected that they were in a higher amount in the fibre-rich fraction, being responsible for higher interferences with the nitrosohaemochrome. 

By contrast, the effect of the storage period upon the reflectance spectrum (% reflectance) of sausages was only significant (*p* < 0.05) in control sausages and only after 670 nm. Reflectance spectra for sausages with quinoa products added (BQS and RFBQS) were not affected by the storage period. 

### 3.2. Residual Nitrite Level

Bologna-type sausages are cured and cooked meat products, which means that they have nitrate/nitrite in their formulation. The amount in which they can be added is subject to official regulation [32,33] because although they have important desirable effects in several aspects of meat products processing and development (mainly colour fixation, fat oxidation, flavour, and microbiological safety) [34,35], their application concerns a potential risk of the formation of carcinogenic, teratogenic, and mutagenic nitroso compounds [36,37], leading to a trend towards their decreasing. As soon as nitrite is added to meat batter, it is reduced to nitric oxide to react with myoglobin and other active compounds to develop all the positive actions that have been previously described. The evolution of residual nitrite levels during storage is shown in Table 3. Sausages with quinoa products added (BQS and RFBQS) showed higher residual nitrite levels than control, with RFBQS showing the highest levels. It is well known that some vegetable extracts have been used as nitrite replacers in several meat products due to their ability to accumulate nitrate in their structure during their growth [38]. Several authors have reported the same behaviour for quinoa seeds [39], which could explain the higher (*p* < 0.05) residual nitrite level in quinoa-added sausages with respect to the control. Residual nitrite levels decreased (*p* < 0.05) significantly and steadily during storage in all the samples but varied among types of bologna-type sausages. Sausages with quinoa products added (BQS and RFBQS) showed higher (*p* < 0.05) residual nitrite levels than control at all measurements time during the storage period, with RFBQS sausages showing the highest values. This behaviour has also been previously reported for other cured meat products with added quinoa [18,40,41]. At the end of storage, all sausages showed a 25–30% reduction with respect to day 0. Similar values and decreased rates in residual nitrite during storage have been reported for bologna-type sausages [26,42,43,44]. The observed decrease in residual nitrite suggests that oxidation–reduction reactions occurred to convert the nitrite to nitrous oxide. In addition, it was postulated that all of the available nitrite was depleted during curing and processing with a subsequent conversion of the sodium nitrate to nitrite during storage. It must be highlighted that the final values of residual nitrites in all bologna-type sausages at all measurement times during the storage period were below those permitted in cooked-cured products (100 mg/kg) [45].

### 3.3. Lipid Oxidation

Lipid oxidation is a major cause of deterioration in the quality of meat and meat products, its importance being even higher in meat products because most of the processing techniques applied (mincing, salting, cooking, among others) favour its development. The extent of lipid oxidation in meat products is commonly measured by the TBARS test. As can be seen in Table 3, TBARS increased significantly during storage for all formulations; it must be noted that sausages with quinoa products added (BQS and RFBQS) showed values lower (*p* < 0.05) than the control for all the days studied. In addition, sausages with quinoa seeds (BQS) showed the lowest TBARS values throughout the entire storage period. This pattern of lipid oxidation could be due to the presence of antioxidant compounds in quinoa seeds [46]. Several studies have been carried out to study the antioxidant compounds and antioxidant activity of several quinoa seeds of different colour varieties (white, red, and black). Pellegrini et al. [41] reported higher total phenolic content and antioxidant activity in flours from black quinoa seeds that that of those of white quinoa. Pereira et al. [6] found that although the 3 isoforms of tocopherols (α, γ, and δ-tocopherol) were detected in the three quinoa colour varieties, with γ-tocopherols being the predominant isoform, the black grains contained the highest concentrations. It must be noted that γ-tocopherol can have equal or even stronger antioxidant properties than α-tocopherol [46]. Differences in TBARS values between BQS and RFBQS samples could be attributed to the degradation of some of these antioxidant compounds during the quinoa processing for the extraction of its fibre-rich fraction. Similar behaviour has been reported in cooked meat products with citrus and pomegranate extracts added, and also, in this case, the antioxidant effect was attributed to the antioxidant compounds (mainly polyphenols) found in these extracts [24]. Although TBARS values were increased during storage time, the amount of malondialdehyde (mg) per kg sample did not exceed the limit for which it could be detected as a rancid taste in sensory analysis for all formulations studied [47].

### 3.4. Microbiological Analysis

**Figure 2:**
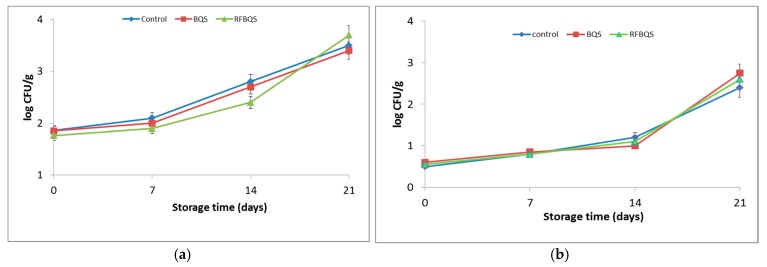
Microorganism, (**a**) mesophilic aerobic bacteria; (**b**) lactic acid bacteria, counts (log colony forming units (CFU)/g) of Bologna-type sausages (control, sausage with black quinoa seeds (BQS), and sausage with its fibre-rich fraction (RFBQS)) during cold storage.

The addition of quinoa products did not significantly affect microbiological growth during the storage of bologna-type sausages (Figure 2). Enterobacteria and moulds and yeast were not detected in all formulations during the 21 days of storage which would indicate good sanitary conditions of the raw material, but also good manufacturing practices, without undervaluing the effect of the thermal processing in this type of meat products. Initial mesophilic aerobic bacteria and lactic acid bacteria counts were very low (<2 log CFU/g and <1 log CFU/g, respectively) without significant formulation-related differences. MAB and LAB growth was observed during storage in all bologna-type sausages. During the 21 days of storage, MAB and LAB count remained below 4 log CFU/g and 3 log CFU/g, respectively, for all treatments, without significant differences between treatments. These microbial counts observed during storage in all treatments (control, BQS, and RFBQS) are considered insufficient to promote any of the characteristics of a degraded product (higher viscosity, colour changes, off-flavours) [2,43] guaranteeing that the product is safe for consumers [48]. Similar microbiological counts during cold storage have been reported by Ranucci et al. [49] in cooked sausages with almond nut and emmer wheat added.

## 4. Conclusions

The results showed that the reformulation of Bologna-type sausages using black quinoa products (either as whole seeds or as a fibre-rich fraction obtained from the quinoa wet-milling process) is a technologically viable alternative for the introduction of quinoa products as ingredients in the meat industry. In addition, sausages with quinoa products added exhibited greater resistance to oxidation than control sausages during chilled storage, without affecting pH or microbiological safety. Although the addition of quinoa products in Bologna-type sausages initially modified some colour parameters, these differences were masked through the storage period without affecting the typical reflectance spectrum shape for this type of meat product. The decrease in the residual nitrite level during storage was not affected by quinoa addition (in either form) although sausages with quinoa added started with higher levels probably due to the stored nitrate in the quinoa seeds. Therefore, black quinoa products can be considered as promising ingredients to be used as antioxidants and natural nitrate sources in Bologna-type sausages.

## Figures and Tables

**Table 1:** Formulation of Bologna-type sausages containing quinoa products (black quinoa seeds (BQS) and the fibre-rich fraction from its wet-milling process (RFBQS)).

Ingredients (%)	Control	BQS	RFBQS
Lean pork	41.4	41.4	41.4
Pork backfat	41.4	41.4	41.4
Salt	2.1	2.1	2.1
Crushed ice	12.1	12.1	12.1
Potato starch	2.5	-	-
Black quinoa seeds	-	2.5	
Fibre-rich fraction from quinoa wet-milling process	-	-	2.5
Sodium tripolyphosphate	0.03	0.03	0.03
Sodium nitrite	0.015	0.015	0.015
Sodium ascorbate	0.042	0.042	0.042
Black pepper	0.16	0.16	0.16
Nutmeg	0.04	0.04	0.04
Garlic	0.083	0.083	0.083
TOTAL	100	100	100

**Table 2:** Physicochemical properties (pH and instrumental colour) during storage of Bologna-type sausages containing black quinoa products.

		Day 0	Day 7	Day 14	Day 21
**pH**					
	**Control**	6.40 ± 0.02 ^aw^	6.42 ± 0.02 ^aw^	6.43 ± 0.02 ^aw^	6.43 ± 0.02 ^aw^
	**BQS**	6.44 ± 0.01 ^aw^	6.45 ± 0.01 ^aw^	6.46 ± 0.02 ^aw^	6.44 ± 0.01 ^aw^
	**RFBQS**	6.42 ± 0.02 ^aw^	6.43 ± 0.01 ^aw^	6.45 ± 0.02 ^aw^	6.44 ± 0.01 ^aw^
L*					
	**Control**	64.10 ± 1.72 ^aw^	64.00 ± 1.02 ^aw^	63.99 ± 1.89 ^aw^	63.66 ± 2.50 ^aw^
	**BQS**	64.25 ± 1.69 ^aw^	64.12 ± 2.02 ^aw^	64.00 ± 1.56 ^aw^	63.88 ± 2.22 ^aw^
	**RFBQS**	58.86 ± 2.00 ^by^	60.56 ± 2.00 ^bx^	61.05 ± 1.11 ^bx^	63.79 ± 2.01 ^aw^
a*					
	**Control**	6.02 ± 0.43 ^aw^	6.25 ± 0.56 ^aw^	5.96 ± 1.14 ^aw^	5.76 ± 0.71 ^aw^
	**BQS**	4.96 ± 0.69 ^bw^	5.22 ± 1.47 ^aw^	5.26 ± 2.00 ^aw^	5.55 ± 0.99 ^aw^
	**RFBQS**	3.08 ± 1.04 ^cx^	3.56 ± 1.23 ^bx^	5.39 ± 2.10 ^aw^	5.58 ± 1.00 ^aw^
b*					
	**Control**	10.12 ± 0.54 ^aw^	9.89 ± 2.00 ^aw^	9.78 ± 1.56 ^aw^	10.02 ± 0.75 ^aw^
	**BQS**	10.22 ± 1.16 ^aw^	9.72 ± 1.10 ^aw^	10.23 ± 1.87 ^aw^	10.02 ± 0.73 ^aw^
	**RFBQS**	7.14 ± 0.80 ^bx^	7.56 ± 1.54 ^bx^	9.59 ± 1.03 ^aw^	9.96 ± 0.78 ^aw^
C*					
	**Control**	11.78 ± 0.65 ^aw^	11.60 ± 0.35 ^aw^	11.45 ± 0.39 ^aw^	11.58 ± 1.00 ^aw^
	**BQS**	11.38 ± 1.22 ^aw^	11.03 ± 1.00 ^aw^	11.50 ± 0.88 ^aw^	11.47 ± 1.10 ^aw^
	**RFBQS**	7.81 ± 1.09 ^bx^	8.35 ± 0.56 ^bx^	11.00 ± 0.99 ^aw^	11.43 ± 1.05 ^aw^
H*					
	**Control**	59.29 ± 1.20 ^cw^	57.67 ± 1.13 ^cx^	58.63 ± 2.08 ^bwx^	60.30 ± 2.23 ^aw^
	**BQS**	64.10 ± 2.99 ^bw^	61.73 ± 2.22 ^bx^	62.73 ± 1.56 ^ax^	61.21 ± 2.33 ^ax^
	**RFBQS**	67.11 ± 5.10 ^aw^	64.74 ± 2.08 ^ax^	60.67 ± 1.39 ^ay^	60.96 ± 3.02 ^ay^
ΔE*					
	**Control**				
	**BQS**	1.07 ^bw^	1.05 ^bw^	0.83 ^bw^	0.31 ^aw^
	**RFBQS**	6.71 ^aw^	4.94 ^ax^	3.00 ^ay^	0.24 ^az^

BQS: sausage with 3% black quinoa seeds; RFBQS: sausage with 3% fibre-rich fraction from the wet-milling of black quinoa seeds. ^a–c^ Different letters in the same column indicate significant differences (*p* < 0.05). ^w–z^ Different letters in the same row indicate significant differences (*p* < 0.05).

**Table 3:** Residual nitrite level and Thiobarbituric acid reactive substances TBARS values during storage of Bologna-type sausages containing black quinoa products.

		**Day 0**	**Day 7**	**Day 14**	**Day 21**
**Residual Nitrite Level (mg NaNO_2_/kg)**					
	**Control**	32.86 ± 1.86 ^cw^	30.76 ± 1.01 ^cw^	24.91 ± 0.85 ^cx^	23.01 ± 0.65 ^cx^
	**BQS**	36.20 ± 0.23 ^bw^	34.36 ± 1.09 ^bw^	30.09 ± 1.22 ^bx^	26.95 ± 0.99 ^by^
	**RFBQS**	43.64 ± 0.21 ^aw^	42.08 ± 0.99 ^aw^	36.71 ± 1.30 ^ax^	32.74 ± 1.20 ^ay^
**TBARS (mg MA/kg)**					
	**Control**	0.44 ± 0.03 ^az^	0.52 ± 0.02 ^ay^	0.59 ± 0.03 ^ax^	0.68 ± 0.01 ^aw^
	**BQS**	0.22 ± 0.03 ^cx^	0.25 ± 0.01 ^cx^	0.29 ± 0.03 ^cw^	0.30 ± 0.02 ^cw^
	**RFBQS**	0.30 ± 0.02 ^bx^	0.33 ± 0.02 ^bx^	0.37 ± 0.02 ^bw^	0.38 ± 0.02 ^bw^

BQS: sausage with 3% black quinoa seeds; RFBQS: sausage with 3% fibre-rich fraction from the wet-milling of black quinoa seeds. Means ± standard deviation. ^a–c^ Different letters in the same column indicate significant differences (*p* < 0.05). ^w–z^ Different letters in the same row indicate significant differences (*p* < 0.05).

## References

[1] De Almeida P.L., de Lima S.N., Costa L.L., de Oliveira C.C., Damasceno K.A., dos Santos B.A., Campagnol P.C.B. (2015). Effect of jabuticaba peel extract on lipid oxidation, microbial stability and sensory properties of Bologna-type sausages during refrigerated storage. Meat Sci..

[2] Fernández-López J., Lucas-González R., Viuda-Martos M., Sayas E., Navarro C., Haros C.M., Pérez-Alvarez J.A. (2019). Chia (*Salvia hispánica* L.) products as ingredients for reformulating frankfurters: Effects on quality properties and shelf-life. Meat Sci..

[3] Seo J.K., Parvin R., Yim D.G., Zahid M.A., Yang H.S. (2019). Effects on quality properties of cooked pork sausages with *Caesalpinia sappan* L. extract during cold storage. J. Food Sci. Technol..

[4] Essien E. (2003). Sausage Manufacture. Principles and Practice.

[5] Gordillo-Bastidas E., Díaz-Rizzolo D.A., Roura E., Massanés T., Gomis R. (2016). Quinoa (*Chenopodium quinoa* Willd), from nutritional value to potential health benefits: An integrative review. J. Nutr. Food Sci..

[6] Pereira E., Encina-Zelada C., Barros L., González-Barron U., Cadavez V., Ferrerira I.C.F.R. (2019). Chemical and nutritional characterization of *Chenopodium quinoa* Willd (quinoa) grains: A good alternative to nutritious food. Food Chem..

[7] Ramos-Díaz J.M., Kirjoranta S., Tenitz S., Penttillä P.A., Serimaa R., Lampi A.M., Jouppila K. (2013). Use of amaranth, quinoa and kañiwa in extruded corn-based snacks. J. Cereal. Sci..

[8] Wang S., Zhu F. (2016). Formulation and quality attributes of quinoa food products. Food Bioprocess Technol..

[9] Ballester-Sánchez J., Gil J.V., Fernández-Espinar M.T., Haros C.M. (2019). Quinoa wet-milling: Effect of steeping conditions on starch recovery and quality. Food Hydrocoll..

[10] Mufari J.R., Miranda-Villa P.M., Calandri E.I. (2018). Quinoa germ and starch separation by wet milling, performance and characterization of the fractions. LWT Food Sci. Technol..

[11] Fernández-Diez A., Caro I., Castro A., Salvá B.K., Ramos D.D., Mateo J. (2016). Partial fat replacement by boiled quinoa on the quality characteristics of a dry-cured sausage. J. Food Sci..

[12] Baioumy A.A., Bobreneva I.V., Tvorogova A.A., Shobanova T.V. (2018). Possibility of using quinoa seeds (*Chenopodium quinoa*) in meat products and its impact on nutritional and organoleptic characteristics. Biosci. Res..

[13] Rizzi G.Z., Pereira T., de Souza E.L., Soares F.A.A.S.M. (2017). Preparation and evaluation of tuscan sausage of chicken meat with the addition of quinoa. Anuário Pesqui. E Extensão Unoesc Videira.

[14] Shokry A.M. (2016). The usage of quinoa flour as a potential ingredient in production of meat burger with functional properties. Middle East J. Appl. Sci..

[15] Bagdatli A. (2018). The influence of quinoa (*Chenopodium quinoa* Willd.) flour on the physicochemical, textural and sensorial properties of beef meatball. Ital. J. Food Sci..

[16] Hleap-Zapata J.I., Rodríguez de la Pava G.C. (2018). Physicochemical analysis of frankfurter type sausages made with red tilapia fillet waste (*Oreochromis* sp.) and quinoa flour (*Chenopodium quinoa* W.). Braz. J. Food Technol..

[17] Verma A.K., Rajkumar V., Kumar S. (2019). Effect of amaranth and quinoa seed flour on rheological and physicochemical properties of goat meat nuggets. J. Food Sci. Technol..

[18] Fernández-López J., Lucas-González R., Viuda-Martos M., Sayas-Barberá E., Ballester-Sánchez J., Haros C.M., Martínez-Mayoral A., Pérez-Álvarez J.A. (2020). Chemical and technological properties of bologna-type sausages with added black quinoa wet-milling coproducts as binder replacer. Food Chem..

[19] Vargas-Zambrano P., Riera-González G., Cruz-Viera L. (2019). Quinoa as gelling agent in a mortadella formulation. Intern. Food Res. J..

[20] Özer C.O., Seçen S.M. (2018). Effects of quinoa flour on lipid and protein oxidation in raw and cooked beef burger during long term frozen storage. Food Sci. Technol..

[21] AMSA (2012). Meat Color Measurement Guidelines.

[22] (1975). ISO/DIS 2918.26, International Standard 2918. Meat and Meat Products: Determination of Nitrite Content. Ref. No. ISO 2918:1975.

[23] Rosmini M.R., Perlo F., Pérez-Alvarez J.A., Pagán-Moreno M.J., Gago-Gago A., López-Santoveña F., Aranda-Catalá V. (1996). TBA test by an extractive method applied to paté. Meat Sci..

[24] Ranucci D., Roila R., Andoni E., Braconi P., Branciari R. (2019). Punica granatum and Citrus spp. extract mix affects spoilage microorganisms growth rate in vacuum-packaged cooked sausages made from pork meat, emmer wheat (*Triticum dicoccum Schübler*), almond (*Prunus dulcis Mill.*) and hazelnut (*Corylus avellana* L.). Foods.

[25] Sakata R. (2000). Studies on physicochemical characteristics of red pigments in meat products. Anim. Sci. J..

[26] Fernández-Ginés J.M., Fernández-López J., Sayas-Barberá M.E., Sendra E., Pérez-Alvarez J.A. (2003). Effect of storage conditions on quality characteristics of Bologna sausages made with citrus fiber. J. Food Sci..

[27] Jongberg S., Torngren M.A., Gunvig A., Skibsted L.H., Lund M.N. (2013). Effect of green tea or rosemary extract on protein oxidation in Bologna type sausages prepared from oxidatively stressed pork. Meat Sci..

[28] Kulkarni S., De Santos F.A., Kattamuri S., Rossi S.J., Brewer M.S. (2011). Effect of grape seed extract on oxidative, color and sensory stability of a pre-cooked, frozen, re-heated beef sausage model system. Meat Sci..

[29] Martínez J.A., Melgosa M., Pérez M., Hita E., Negueruela A.I. (2001). Visual and instrumental color evaluation in red wines. Food Sci. Technol. Intern..

[30] Liu Y., Chen Y.R. (2001). Analysis of visible reflectance spectra of stored, cooked and diseased chicken meats. Meat Sci..

[31] Gandía-Herrero F., García-Carmona F. (2013). Biosynthesis of betalains: Yellow and violet plant pigments. Trends Plant. Sci..

[32] (2014). EC, Commission Regulation (EU) No 601/2014 of 4 June 2014 amending Annex II to Regulation (EC) No 1333/2008 of the European Parliament and of the Council as regards the food categories of meat and the use of certain food additives in meat preparations. Off. J. Eur. Union.

[33] (2010). EFSA, EFSA panel on food additives and nutrient sources added to food (ANS); Statement on nitrites in meat products. EFSA J..

[34] Andrée S., Jira W., Schwind K.H., Wagner H., Schwägele F. (2010). Chemical safety of meat and meat products. Meat Sci..

[35] Chetty A.A., Prasad S., Castro-Pinho O., Medeiros de Morais C. (2019). Estimated dietary intake of nitrate and nitrite from meat consumed in Fiji. Food Chem..

[36] Santarelli R.L., Pierre F., Corpet D.E. (2008). Processed meat and colorectal cancer: A review of epidemiologic and experimental evidence. Nutr. Cancer.

[37] Viuda-Martos M., Fernández-López J., Sayas-Barbera E., Sendra E., Navarro C., Pérez-Álvarez J.A. (2009). Citrus co-products as technological strategy to reduce residual nitrite content in meat products. J. Food Sci..

[38] Hmelak-Gorenjak A., Cencic A. (2013). Nitrate in vegetables and their impact on human health. A review. Acta Aliment..

[39] Gutiérrez-Larrazabal A., Soto-Hernández M., López-Castañeda C., Mendoza-Martínez G.D., García-Velázquez A., Mendoza-Castillo M.A. (2004). Nitrates, oxalates and alkaloids in two phenological stages of quinoa (Chenopodium quinoa Willd) in irrigated and rainfed conditions. Rev. Fitotec. Mex..

[40] Escamez A., Viuda-Martos V., Fernández-López J., Sayas-Barberá
 E., Navarro C., Martínez-Mayoral A., Pérez-Alvarez J.A. ¿Quinoa (*Chenopodium quinoa)*, un nuevo ingrediente para los productos cárnicos crudo-curados?. Proceedings of the IX Congreso CyTA-CESIA.

[41] Pellegrini M., Lucas-González R., Sayas E., Fernández-López J., Pérez-Alvarez J.A., Viuda-Martos M. (2018). Quinoa (*Chenopodium quinoa* Willd) paste as partial fat replacer in the development of reduced fat cooked meat product type pâté: Effect on quality and safety. CyTA J. Food.

[42] Hill L.H., Webb N.B., Moncol L.D., Adams A.T. (1973). Changes in residual nitrite in sausage and luncheon meat products during storage. J. Milk Food Technol..

[43] Viuda-Martos M., Ruiz-Navajas Y., Fernández-López J., Pérez-Álvarez J.A. (2010). Effect of orange dietary fibre, oregano essential oil and packaging conditions on shelf-life of bologna sausages. Food Control..

[44] Viuda-Martos M., Ruiz-Navajas Y., Fernández-López J., Pérez-Álvarez J.A. (2010). Effect of added citrus fibre and spice essential oils on quality characteristics and shelf-life of mortadella. Meat Sci..

[45] (2003). EFSA, Opinion of the scientific panel on biological hazards on the request from the Commission related to the effects of nitrites/nitrates on the microbiological safety of meat products. EFSA J..

[46] Tang Y., Li X., Zhang B., Chen P.X., Liu R., Tsao R. (2015). Characterisation of phenolics: Betanins and antioxidant activities in seeds of three *Chenopodium quinoa* Willd genotypes. Food Chem.

[47] Ockerman H.W. (1976). Quality Control of Post Mortem Muscle and Tissue.

[48] ICMSF (1986). Microorganisms in Foods 2. Sampling for Microbiological Analysis: Principles and Specific Applications.

[49] Ranucci D., Miraglia D., Branciari R., Morganti G., Roila R., Zhou K., Braconi P. (2018). Frankfurters made with pork meat, emmer wheat (*Triticum dicoccum Schübler*) and almonds nut (*Prunus dulcis* Mill.): Evaluation during storage of a novel food from an ancient recipe. Meat Sci..

